# Tailored 3D Lattice Microstructures for Enhanced Functionality in Blood‐Gas Exchange

**DOI:** 10.1002/advs.202501162

**Published:** 2025-04-17

**Authors:** Kai P. Barbian, Teresa Lemainque, Ina Grunden, Roman Iwa, Bettina Wiegmann, John Linkhorst, Matthias Wessling, Jan Heyer, Ulrich Steinseifer, Michael Neidlin, Sebastian V. Jansen

**Affiliations:** ^1^ Department of Cardiovascular Engineering Institute of Applied Medical Engineering Medical Faculty RWTH Aachen University Forckenbeckstr. 55 52074 Aachen Germany; ^2^ Department of Diagnostic and Interventional Radiology Medical Faculty RWTH Aachen University 52074 Aachen Germany; ^3^ Department for Cardiothoracic Transplantation and Vascular Surgery Hannover Medical School Carl‐Neuberg‐Straße 1 30625 Hannover Germany; ^4^ Implant Research and Development (NIFE) Lower Saxony Center for Biomedical Engineering Stadtfelddamm 34 30625 Hannover Germany; ^5^ German Center for Lung Research (DZL) Carl‐Neuberg‐Straße 1 30625 Hannover Germany; ^6^ Chemical Process Engineering (AVT.CVT) RWTH Aachen University Forckenbeckstr. 51 52074 Aachen Germany; ^7^ Process Engineering of Electrochemical Systems Technical University of Darmstadt Otto‐Berndt‐Str. 2 64287 Darmstadt Germany; ^8^ DWI – Leibniz‐Institute for Interactive Materials Forckenbeckstr. 50 52074 Aachen Germany

**Keywords:** additive manufacturing, design optimization, flow homogeneity, lattice structures, membrane oxygenators, structure adaptation, tpms

## Abstract

Current membrane oxygenators for extracorporeal life support (ECLS) are facing their limits regarding gas exchange efficiency and long‐term stability. One aspect adding to these limitations is inhomogeneous blood flow distribution inside the oxygenator's membrane structure. Triply periodic minimal surface (TPMS) lattice structures are proposed to provide increased mass transfer efficiency and local adaptability introducing heterogeneous properties. However, the adaptation of these structures for blood flow, as in ECLS, is challenging as a hemocompatible flow distribution must be established. In this study, this study proposes a novel method for the smooth, multi‐scale modification of TPMS lattice structures creating a tailored flow distribution suited for blood‐gas exchange. It implements this method into an automatic structure optimization within an oxygenator. After manufacturing prototypes, it experimentally evaluate the 3D flow distribution using time‐resolved, contrast enhanced computed tomography comparing the optimized structure to reference geometries. The TPMS structure modification provides a significant change in flow distribution, improving homogeneity by up to 12%. The approach to creating tailored 3D TPMS lattice structures can be directly transferred to various other applications in the field of heat and mass transfer to enhance functionality, e.g., for heat exchangers or membrane contactors.

## Graphical abstract

Common hollow fiber structures are associated with inhomogeneous flow, leading to many complications in the medical application of extracorporeal membrane oxygenation. Using locally adapted lattice structures, the overall flow distribution can be manipulated. As validated by computed tomography, this modification leads to a major enhancement of flow homogeneity to increase hemocompatibility and mass transfer efficiency.

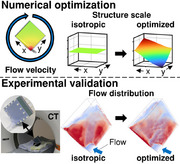

## 1 Introduction

Severe acute and chronic respiratory diseases are the third leading cause of death worldwide.^[^ ^1^, ^2^ ^]^ One treatment option is extracorporeal membrane oxygenation. Here, oxygen is transferred into the blood while carbon dioxide is removed outside of the human body. Despite its life‐saving potential, ECMO remains a last resort option because of a multitude of complications resulting in up to 70% mortality rate.^[^ ^3^, ^4^, ^5^ ^]^ Suboptimal flow paths and a large foreign surface area in contact with blood inside the oxygenator can lead to complications like thrombus formation or systemic inflammation. To avoid this thrombus formation, high level anticoagulation is needed which can lead internal bleeding events.^[^ ^6^, ^7^, ^8^ ^]^

Membrane structures based on triply periodic minimal surface (TPMS) lattices have been proposed for the use in oxygenators, as they can provide increased mass transfer efficiency and show a lower tendency for blood clot formation, compared to the current hollow fiber membrane (HFM) technology.^[^ ^9^, ^10^, ^11^, ^12^ ^]^ This is achieved through a change in microscopic flow structure (cf. Figure S7, Supporting Information). Moreover, heterogenous TPMS lattice structures have been demonstrated to provide improved functionality for a variety of technical applications. Due to a grading of the structure's geometry, its characteristics can be adapted for specific applications.^[^ ^13^, ^14^ ^]^ This local adaptation of lattice structure geometry has already been used in other biomedical applications, such as bone implants or scaffolds for tissue regeneration.^[^ ^15^, ^16^, ^17^ ^]^ However, its implementation into fluid flow applications, like membrane structures for oxygenators, is much less studied as specific challenges must be addressed. Structure adaptation maintaining the smooth transitions required for blood flow is a challenge. Abrupt changes of the surface structure or highly skewed channel geometry would introduce the risk of blood clotting due to flow stagnation.^[^ ^18^, ^19^ ^]^ In addition, physical blood damage can be caused by increased shear rates.^[^ ^20^, ^21^ ^]^ The digital processing and manufacturing of such complex structures where its microscopic features differ from its outer dimensions by multiple orders of magnitude poses a major challenge due to the large amount of geometric data. A ready‐to‐use solution for the data processing of such a geometric entity is not available. Finally, a comprehensive evaluation of the fluid flow patterns inside lattice structure geometries is missing.

**Figure 1:**
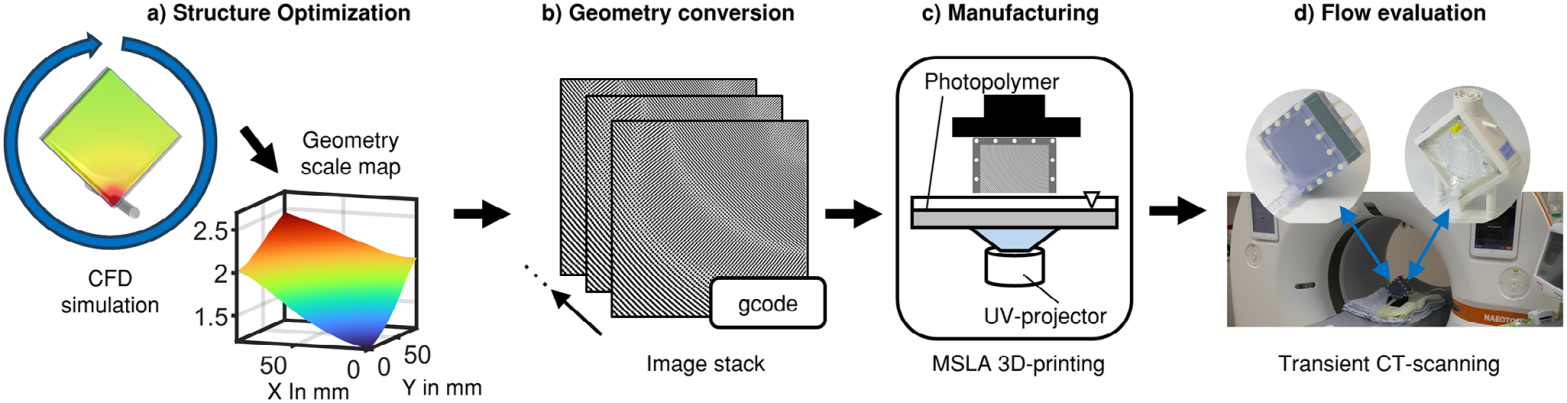
Conducted steps for the creation and experimental investigation of TPMS lattice structures. a) Structure optimization strategy, b) geometry conversion into physical representation, c) manufacturing with 3D‐printing, d) flow evaluation with time resolved CT scans.

To overcome these challenges, we developed a novel method to create adapted TPMS lattice structures for optimal flow patterns. We propose a local scaling of the TPMS geometry through coordinate‐transformations for smooth lattice modification. Therefore, we implemented an automatic optimization algorithm that creates an anisotropic TPMS structure yielding homogenous flow inside an oxygenator (**Figure** **1**a). Making use of implicit geometry representations (Figure 1b), we converted the designed TPMS lattice structures into laboratory prototypes by 3D printing (Figure 1c). To finally demonstrate the effect of local TPMS lattice structure adaption, we examined the 3D flow distribution inside the optimized structure, a reference geometry and a commercial oxygenator by performing time resolved, contrast agent (CA) enhanced computed tomography (CT) with a state‐of‐the‐art photon‐counting scanner (Figure 1d). The resulting data also allowed validation of the predicted flow distributions that resulted from the numerical model after structure optimization. Our method for the creation of specifically tailored TPMS lattice geometry can be directly transferred to the manufacturing of gas permeable membranes.

## 2 Results

### 2.1 Structure Optimization

**Figure 2:**
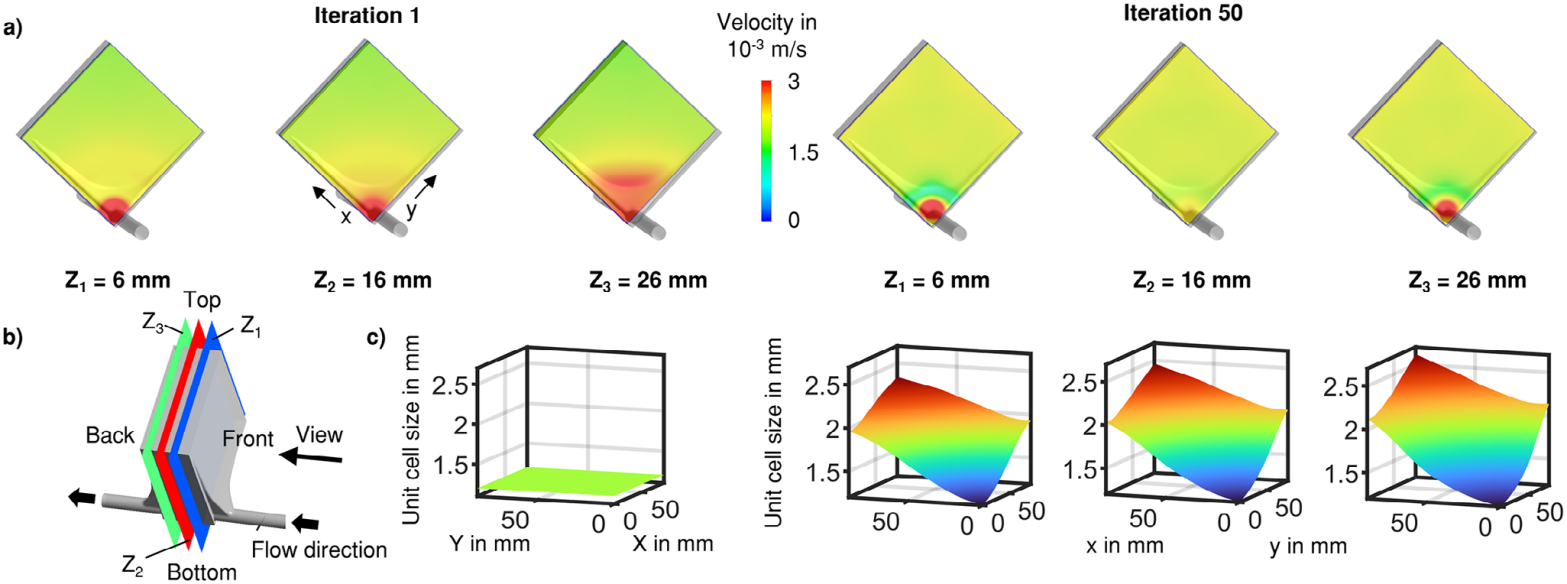
Flow alteration during optimization process. a) Fluid velocity distributions at different Z locations before (left) and after (right) automatic structure optimization, b) definition of Z‐locations of visualization planes, c) respective TPMS unit cell size fields.

TPMS lattice structures for use in blood‐flow applications, such as oxygenators, must fulfil specific requirements. First, a smooth surface without abrupt transitions must be established to avoid blood stagnation areas. Second, a sufficiently large surface area should be provided that is accessed from one single inlet. And third, homogenous overall perfusion is required to maximize mass transfer across the whole membrane surface available. To meet these requirements, we propose a method for locally adjusting the TPMS structure geometry that is based on a coordinate‐transformation. Therefore, an optimal configuration of local geometry scales had to be identified. The process of computational fluid dynamics (CFD) based TPMS structure optimization is shown in **Figure** **2**, where the optimization goal was to yield a uniform flow velocity throughout the structure. Starting from an isotropic TPMS structure, the initial flow velocity for three cut planes (Z_1_‐Z_3_) is shown in Figure 2a with a clear non‐homogeneous flow velocity distribution. In Figure 2b, the locations of the cut planes are illustrated. For TPMS lattices, their unit cell size is a specific parameter that describes the scale of its unique base geometry. Within our algorithm, the local TPMS unit cell size was used as a control variable that defines the structure's geometry. From an initially constant, small TPMS unit cell size field, the optimization algorithm has created a unit cell size distribution that is variable in all three spatial dimensions (Figure 2c). The optimizer showed stable behavior with an asymptotic decrease in change of local unit cell sizes per iteration without the need to introduce additional stabilization strategies. The minimal unit cell size of 1.2 mm from the initialization, that was defined as a lower constraint because of manufacturability, was kept at the bottom of the lattice structure. In the upper regions, the unit cell size was increased by the optimization algorithm (Figure 2c). The maximum unit cell size that was created is located at the top of the structure on its backside (Figure 2c,Z_3_) with a value of 2.8 mm. In a planar view, perpendicular to the flow direction, the largest gradient of unit cell sizes can be observed. However, an increase in unit cell size (e.g., from 2.3 to 2.8 mm in the top corner) was also created from the front to the backside of the lattice structure (Figure 2c). During the optimization, the surface area inside the structure changed from 0.98 m^2^ to 0.73 m^2^. The optimization results yield a much more homogenous, stationary velocity distribution, compared with the initial simulation (Figure 2a).

### 2.2 Manufacturing

**Figure 3:**
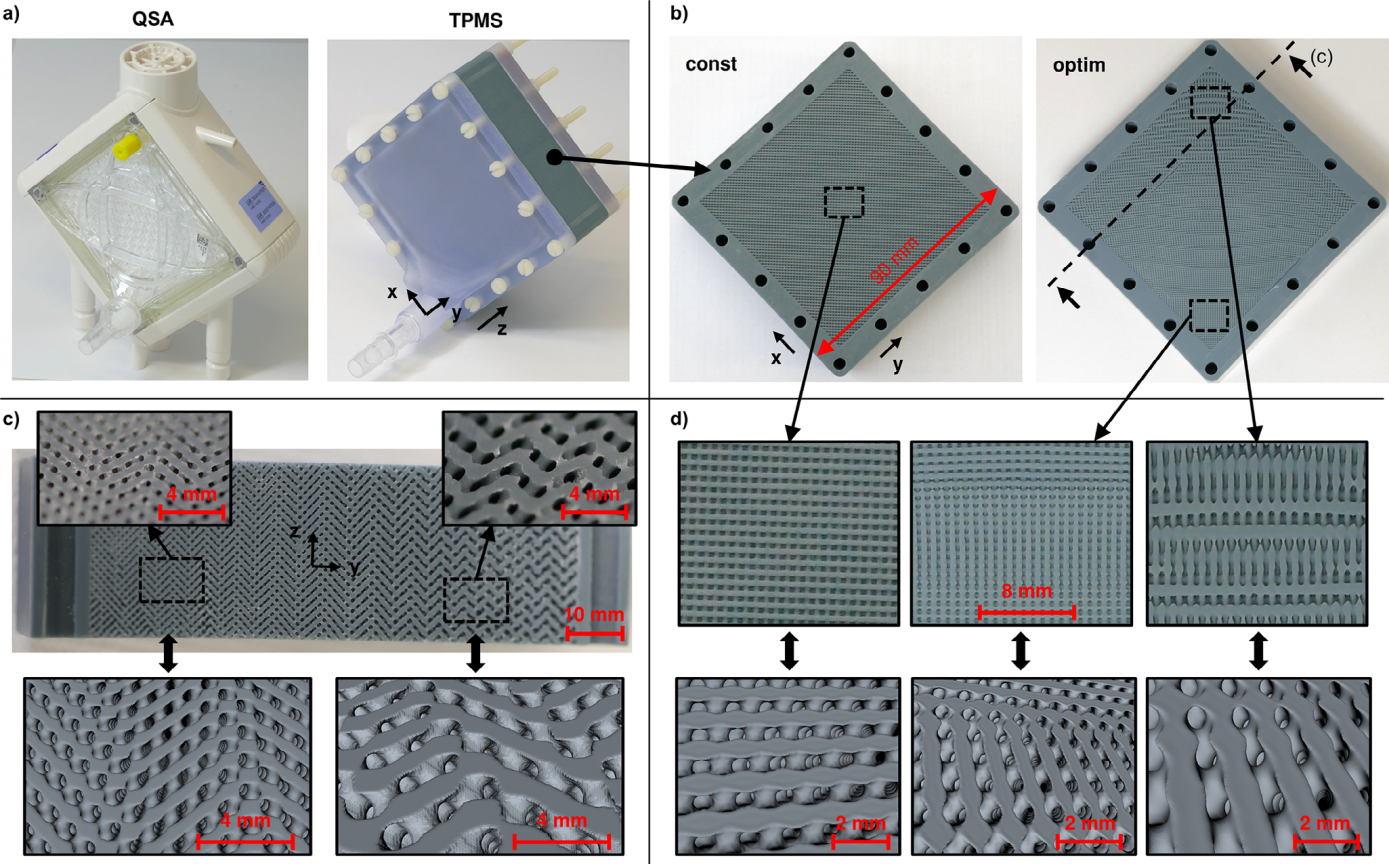
a) Manufactured TPMS prototypes and commercial reference oxygenator (QSA), b) constant (const) and optimized (optim) TPMS lattice geometries, c) close‐up view of cut open cross section (cut along dashed line in section b) of the optimized TPMS structure with respective 3D model representations, d) detail views of local areas from TPMS structures (equal scale bar for all three photographic images) with respective 3D model representations.

The manufactured prototype of the TPMS oxygenator that consists of identical in‐ and outlet diffusers and the 3D printed structure is displayed in **Figure** **3**a alongside a commercial oxygenator (Quadrox‐i Small Adult “QSA”, Maquet Cardiopulmonary, Germany) that was tested as a reference. In Figure 3b, the two types of TPMS lattice structures that were manufactured are shown. The structure with a constant (const) unit cell size shows a regular flow channel geometry in the detail views (Figure 3d). The numerically optimized (optim) structure contains a larger unit cell size at the top and a smaller unit cell size at the bottom. This geometry of the optimized structure is the result of the translation of the unit cell size field from the optimization process into a continuous TPMS geometry. The central cross section cut parallel to the y‐z‐plane (Figure 3c), reveals a continuous and smooth transition from small to large unit cell sizes in y‐axis direction. As shown in the 3D model representations in Figure 3c,d, the basic morphology of the TPMS structure remains constant but is scaled in size. The difference in appearance comes from the different locations where the slicing through the structure was performed and from the continuous shift in TPMS phase to realize its scaling.

### 2.3 3D Flow Field

**Figure 4:**
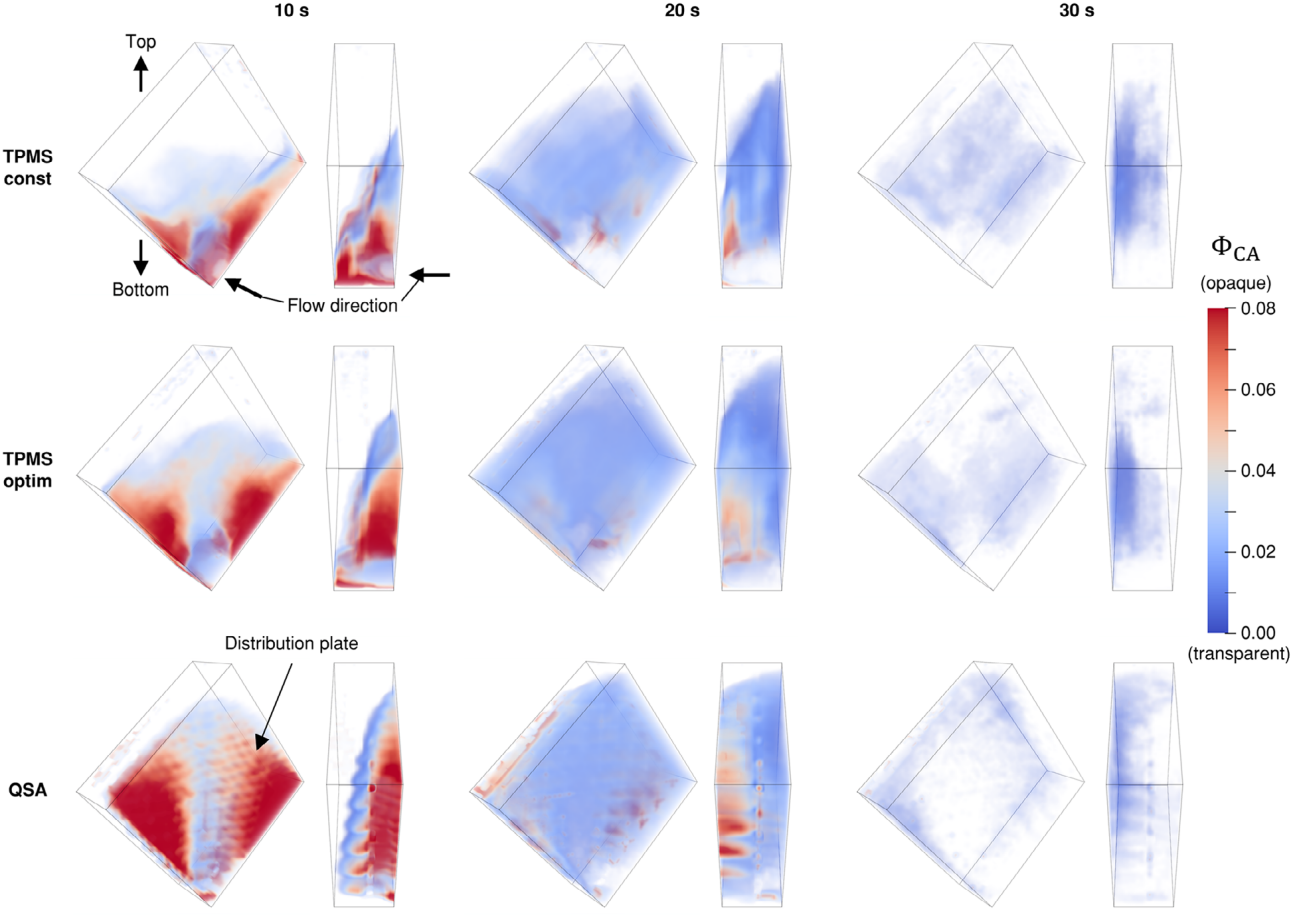
Spatial CA concentration distributions at different timesteps for all test specimens from the experimental CT measurements at a flow rate of 0.5 l/min.

In **Figure** **4**, the spatial distribution of CA is visualized at different time points. All structures show a peak of CA concentration close to the inlet at 10 s after the start of the scan. In the constant TPMS structure at 10 s, the CA is present only in the bottom half of the structure. Inside the optimized structure, the CA is introduced further up toward the top corner. Within the QSA, a CA distribution is observed which proceeds even further upwards. This is because in the QSA a distribution plate is present, specifically designed for the purpose of spreading the flow equally before entering the HFM section. The pattern of holes inside this distribution plate is also visualized by the CA in the experimental results. At 20 s, a similar distribution inside all three specimens can be observed with the constant TPMS structure having the least, the optimized structure having an improved, and the QSA showing the strongest perfusion of the top corner that is furthest away from the inlet. At 30s, the CA has almost left the structures completely. In the TPMS structures, CA is still present in the bottom center region with the constant structure showing more residual CA overall. Inside the QSA, the residual CA is dominantly located in the corner regions, especially inside the top corner, that is mostly free of CA in the TPMS structures. The relative difference of perfusion between the three different structures was observed to be similar also at a higher flow rate of 1.0 l min^−1^, meaning that the flow distribution is independent of the magnitude of flow as long as a Darcy flow regime is predominantly present inside the oxygenator, which is typically the case (cf. Figures S1 and S3, Supporting Information). Figure S2 (Supporting Information) provides the flow visualization for additional timesteps.

**Figure 5:**
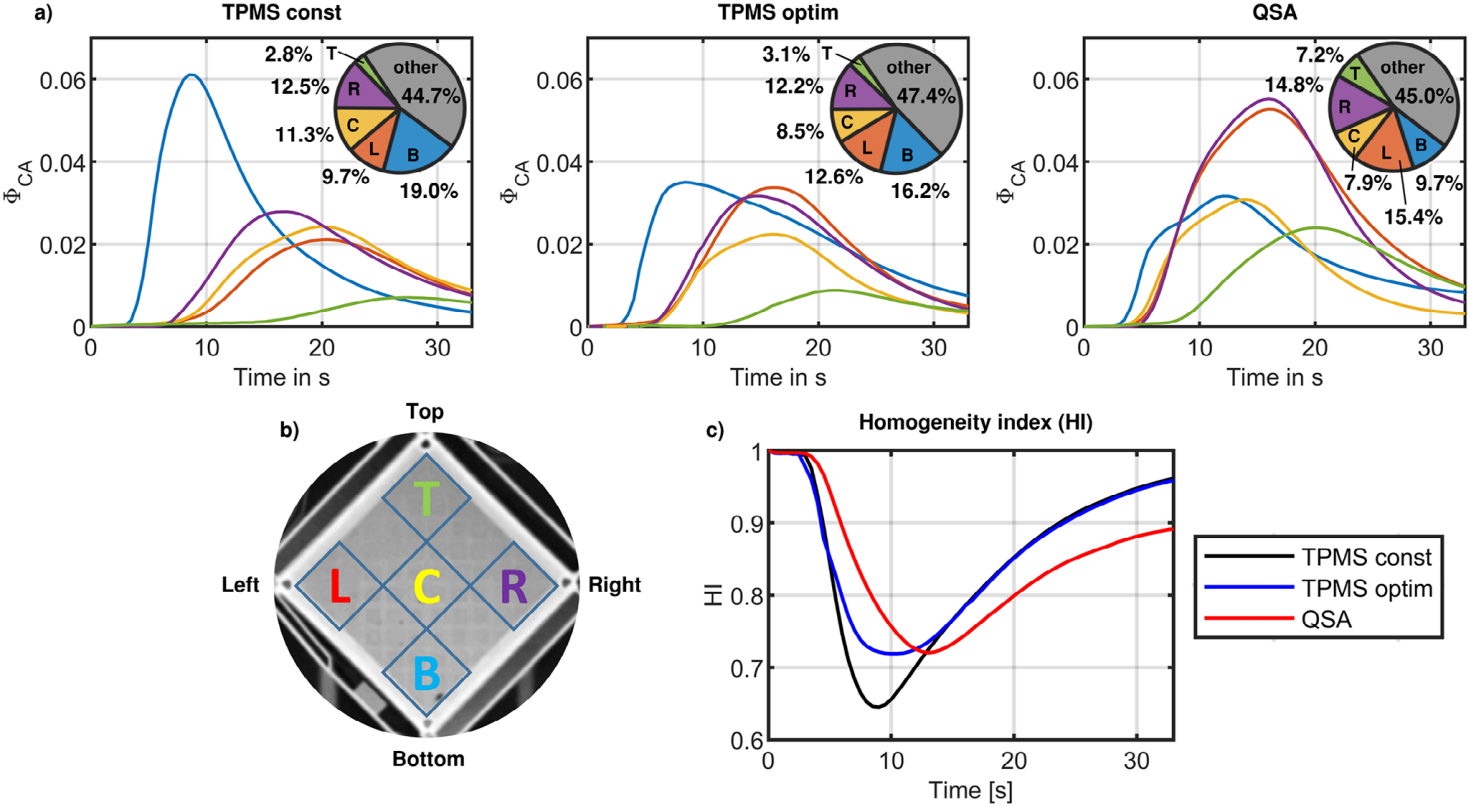
a) Region‐averaged CA concentrations from experimental measurements and respective distributions of CA quantity that has passed through each individual region, visualized in pie charts. Blue line: CA concentration in bottom corner area, red line: left corner area, yellow line: center area, purple line: right corner area, green line: top corner area. b) definition of investigated regions inside the QSA and TPMS lattice structure, bottom (B), left (L), center (C), right (R) and top (T) region. c) Quantitative Homogeneity Index curves over time for the different test specimens at 0.5 l/min flow rate. Black line: TPMS structure with constant unit cell sizes (const), blue line: TPMS structure with optimized unit cell size distribution (optim), red line: commercial reference oxygenator (QSA).

In **Figure** **5**a, the region‐averaged concentrations of CA over time are displayed for all specimens with the definition of individual regions shown in Figure 5b. For the constant TPMS structure, the curve of the bottom corner (B), where in‐ and outlet are located, shows the highest peak of CA concentration which also occurs at first in time. The curves of the central (C) and the left‐ and right corner regions (L, R) have their peak at approximately the same time with also a similar amplitude being half the value of the bottom corner's amplitude. The CA concentration curve for the upper corner (T) shows the lowest amplitude, which occurs almost at the end of measurement time at 28 s. When looking at the optimized TPMS structure, the amplitude of the bottom corner's concentration curve (B) is considerably lower than that of the constant TPMS structure being only slightly higher than the amplitudes of the other regions. Moreover, the peaks of the other regions’ curves occur slightly earlier than in the constant structure, indicating that more flow is directed from the inlet to the upper regions. The amplitudes of the curves for the bottom and left and right corner regions (B, L and R) are all similar in height, showing a more homogenous distribution from the inlet channels into the structure. For the QSA, the CA concentration curves of the right and left corner regions (L and R) show the highest amplitude, which is significantly larger than in the other regions. Here, the peak of the bottom corner region (B) is also the first to occur, directly followed by the central region's peak (C). Inside the QSA, more CA is transferred into the top corner (T), indicated by a larger peak in comparison to the TPMS structures. However, this curve of the top corner (T) also shows the largest value at the end of measurement time, showing that in this region, the CA has the highest residual time of all regions and test specimens. When integrating the CA concentration curves over time, the relative amount of CA that has passed through each region can be calculated. The respective values are provided in Figure 5a in the pie diagrams. In the constant TPMS structure, most of the CA passes through the lower regions B and C with some asymmetry between regions L and R. In the optimized TPMS structure, more CA is transferred from the bottom (B) to the upper regions, especially the right and left corner, to where the flow is guided by the inlet distribution channels. Moreover, an increase in perfusion of the top corner (T) is observed. In the QSA, most CA flows through regions R and L with the remaining CA being equally distributed between the bottom (B), center (C) and top (T) region.

Looking at the Homogeneity Index (HI) further supports this observation trend of flow distribution between the specimens in a more condensed, quantitative way (Figure 5c). The optimized TPMS structure and the QSA show a similar decline in flow homogeneity to a value of 72% with the curves minimum of the optimized TPMS structure occurring 3 s earlier. The HI curve of the constant TPMS structure shows a larger decline to a HI value of 64%, indicating a poorer overall flow homogeneity. However, at the end of measurement time, both HI values of the TPMS structures increased to ≈0.96, close to 1. Here, the value of the QSA is still lowered to a value of 0.89, showing that some non‐equally distributed CA is still present inside the QSA.

### 2.4 Validation of Simulation Model

**Figure 6:**
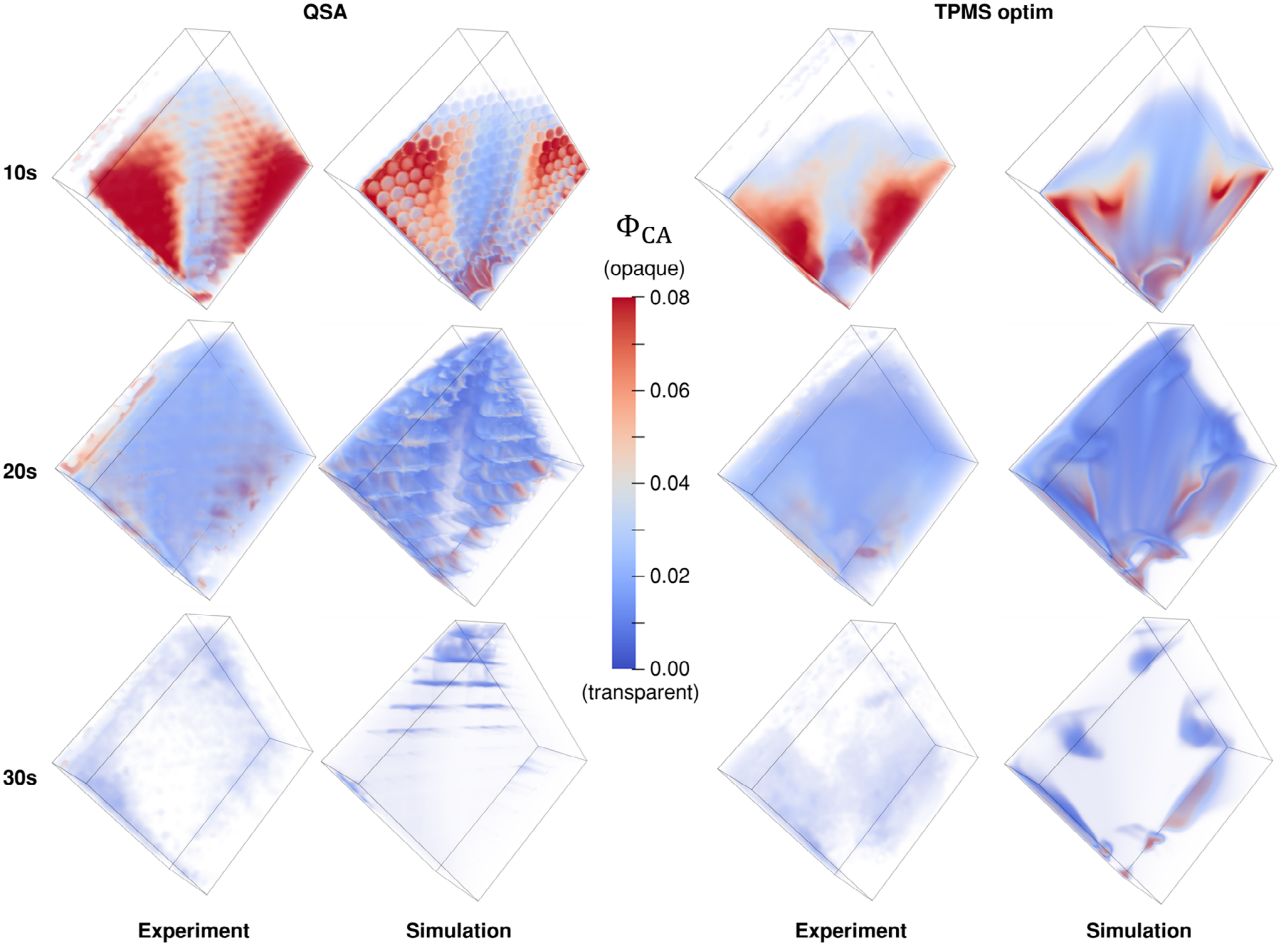
Qualitative comparison of spatial CA concentration distributions from numerical simulation model and experimental measurements at different time steps for the commercial reference oxygenator (left) and the optimized TPMS structure (right).

**Figure 7:**
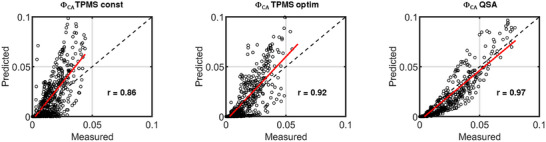
Correlation plots and Spearman's rank coefficient (r) of region averaged CA concentration in all time points for measurements and predicted values from CFD simulation (9849 sample points, *p* < 0.001).

**Figure** **6** shows the spatial distribution of CA from the experimental investigation and the CFD simulations in comparison. A qualitative analysis yields a good agreement between simulation and measurement. Within the simulation, the experimental data of CA movement through the different structures are accurately replicated. However, some differences in the extent of CA concentration in the different regions are visible when looking at the distribution in more detail. The quantitative accuracy of the numerical simulations, which have been used for the optimization process, was evaluated using correlation plots (cf. **Figure** **7**). Here, one single point in the plot corresponds to the averaged CA concentration inside a specific region at a certain time point.

Strong and very strong correlations between simulation and experiment can be observed with Spearman's rank coefficients between 0.86 and 0.97, respectively.^[^ ^22^, ^23^ ^]^ The highest prediction accuracy of the numerical model is achieved for the QSA. The lowest accuracy is observed for TPMS const. Discrepancies between simulation and measurement come from a difference in temporal prediction of the peak of CA concentration in each region, spatial prediction of the pathway of CA and a difference in magnitude of CA concentration. The individual CA distribution curves for specific regions of the different structures that were predicted from the numerical model are provided in the Figure S5 (Supporting Information).

## 3 Discussion

### 3.1 Summary

TPMS lattice structures have been proposed for the design of membrane structures for blood gas transfer as they provide specific advantages for overcoming limitations in ECMO.^[^ ^9^, ^10^, ^11^ ^]^ However, little work has been performed on the integration of those structures being specifically adapted for membrane oxygenators in the required scale. In other biomedical applications, the feature of local geometry anisotropy has been already demonstrated as it can be used to mimic the heterogeneity of natural, biological structures.^[^ ^24^, ^25^, ^26^ ^]^ However, transferring this idea to fluid flow applications, specific challenges must be addressed. Here, a method for local geometry adaptation is required that maintains a smooth and continuous global structure without stagnation regions that would introduce adverse events like blood clotting. The aim of this study was to implement a suitable methodology for the automated local scaling of TPMS lattice structures for the application in membrane oxygenators. Moreover, we aimed for the experimental visualization of the 3D flow distribution inside these structures and the assessment of flow homogeneity.

In this work, we propose a new methodology for the local scaling of TPMS structures by coordinate‐transformation. This approach yielded a smooth, continuous surface inside the TPMS lattice structure that is demanded for the application in membrane oxygenators. The favorable TPMS unit cell base geometry is retained and only scaled locally in size, maintaining microscopic flow characteristics (cf. Figure S7, Supporting Information). This local scaling allowed the manipulation of local flow resistances and therefore the overall, macroscopic flow distribution. With the automated optimization algorithm, a 3D unit cell size field was created that led to a markedly more homogenous flow distribution in steady state simulations. For the experimental investigation of the flow field inside the lattice structures, we introduced time resolved, contrast enhanced, CT scans as a methodology to visualize the underlying flow patterns inside oxygenator membrane structures that are normally inaccessible for investigation. The increased flow homogeneity in the optimized structure, that was predicted by the numerical simulations of the optimization algorithm, was also confirmed by the experiments. This gives proof that a local variation of lattice structure geometry, using our coordinate‐transformation based method, is a powerful tool to tailor the flow distribution inside oxygenator membrane structures to specific needs. The perfusion of the top corner of the optimized TPMS structure, which is most critical, still lacks behind the one inside the QSA. However, flow diverting baffles were used here to improve the flow path design. These baffles, being a passive surface in blood contact without gas exchange, have been reported to promote thrombus adhesion in clinical application.^[^ ^27^ ^]^ Such additional components are not needed for an adapted TPMS membrane structure and a similar perfusion result was already achieved within this study. By replicating the experimental setup with numerical simulations, we were able to validate the accuracy of commonly used porous medium CFD approaches for the prediction of flow fields inside membrane structures. This numerical approach has also been embedded in the optimization algorithm and has been thereby proven valid for this application. Our results show a good overall agreement between the prediction of the simulations and the experimental results. However, when looking into the detail of the flow distribution at certain regions of the structures, there is still some discrepancy visible. In the simulations, many simplifications must be made to simulate the flow field inside such geometrically complicated structures with reasonable computational effort. Most importantly, deviations from the ideal geometry that originate from manufacturing tolerances cannot be fully measured and implemented into the simulations. Moreover, the microscopic flow pattern inside the structures is not fully resolved within the porous medium approach, which can leave out important information of microscopic flow features. Based on these results, the porous medium simulations from the optimization algorithm can be a good predictor for the flow field inside oxygenators in the design process. However, because of the limitations mentioned, they cannot completely replace the experimental investigation of flow homogeneity inside the devices before clinical application, yet. Only the combination of simulation and experimental visualization of the flow field inside the membrane structure can give a complete picture for the structure optimization.

### 3.2 Relation to Other Works

Adapted TPMS lattice structures have been proposed for a wide range of technical applications and scientific fields.^[^ ^13^, ^14^ ^]^ In some other works, heat and mass transfer properties of basic TPMS lattice shapes have been investigated.^[^ ^28^, ^29^, ^30^ ^]^ However, the effect of structure adaptation in larger TPMS lattices on convective fluid flow has been much less studied.^[^ ^13^ ^]^ Concerning biomedical applications, locally adapted lattice structures have been investigated to be used for individualized bone implants or nutrient transport in scaffolds that promote cell growth for tissue regeneration.^[^ ^26^, ^31^, ^32^, ^33^ ^]^ Müller et al. have integrated their method for the local adaption of the mechanical lattice properties into an topology optimization algorithm, similar to our work.^[^ ^34^ ^]^ Also, Hesselmann et al. have automatically optimized local TPMS lattice structure properties for homogenous flow in artificial lungs.^[^ ^35^ ^]^ However, in these approaches the local variation of lattice structure properties is commonly achieved by either stitching regions of different geometry or by manipulation of parameters inside the implicit TPMS equation. The first method is not fully suitable for blood gas transfer applications because it leaves undercuts and cavities that would cause hemostasis. The latter method commonly makes use of a normal shift of the TPMS or of a scaling factor for TPMS periodicity.^[^ ^13^ ^]^ Despite its functionality, the scaling of flow channels by a surface normal shift only provides a limited range for geometry adaptation that restricts the possibility for fully tailored flow distributions. TPMS grading through a periodicity scaling factor is similar to our proposed approach. However, this method usually leads to highly distorted unit cells that would negatively influence flow field and flow predictability. Our more holistic approach of a coordinate‐transformation facilitates the precise control over local geometry features across multiple scales, maintaining a smooth lattice geometry. The manufacturing of large, graded TPMS lattice structures has been demonstrated in other works.^[^ ^14^ ^]^ But within this manuscript, we have manufactured TPMS lattice structures where the relation of overall dimensions to microscopic features is in O(10^4^), the highest presented to date. Maintaining microscopic geometry features inside a structure with dimensions of hundreds of millimeters was necessary for measuring the alteration of macroscopic fluid flow distribution. With our coordinate‐transformation based approach for the local manipulation of TPMS lattice structure scale, we provide a versatile, fully 3D possibility to tailor a lattice structure's performance to specific applications. In our work, the optimization and adaptation of the TPMS lattice structure was specifically developed for the use in membrane oxygenators. Therefore, most of its advantages are provided in the field of fluid flow applications, like blood gas transfer, dialysis, nutrient transport in scaffolds or bioreactors. Here, a smooth flow channel surface is achieved while manipulating fluid flow due to local geometry adjustment. However, this method can also be transferred to other applications, like bone implant design, as a change in TPMS unit cell scale also results in a change in mechanical properties.^[^ ^32^ ^]^ Commonly, flow homogeneity in membrane modules is investigated using dye‐based methods that register the dye concentrations over time at the in‐ and outlet of the structures.^[^ ^35^ ^]^ Within this study, we have visualized the flow distribution inside a complex lattice structure with spatial resolution which provides full insight into the prevailing phenomena. To the best of our knowledge, the influence of adapted TPMS lattice structures on 3D convective fluid flow has never been demonstrated before.

### 3.3 Limitations

As we could show, our CFD‐based structure optimization algorithm yields a homogenous flow velocity field inside the TPMS structure. However, limitations and improvement potential exist for the CFD‐based structure optimization and the experimental flow visualization. The former still uses steady state CFD simulations and only takes the influence of the local TPMS geometry on the local flow velocity at the same spot into account. One approach to consider the effect of local structure adaptation on the overall flow field would be the use of adjoint solvers.^[^ ^36^ ^]^ Moreover, more performance parameters of the oxygenator, like gas transfer or local shear stresses, can be included into the optimization process using adjoint‐based methods. During the optimization process, the total surface area inside the structure was reduced, which could negatively affect the overall mass transfer. However, when there is no lower limit of unit cell size that must be respected, a simultaneous increase and decrease in the structure's scall would happen in different regions, keeping the total surface area constant. Our approach to the local adjustment of TPMS unit cells allows their manipulation across a wide range of scales. Nevertheless, the gradient of unit cell size transition must be limited to not create a distorted geometry. This has been accounted for by using radial basis functions to smooth out the TPMS unit cell size field. The experimental investigation of flow homogeneity was performed using a two‐phase flow with water and CA. The difference in density between these two fluids leads to buoyancy with the CA migrating to the bottom of the oxygenator. Therefore, a shift of the perfusion to the top of the oxygenator is expected when used with blood, even further improving flow homogeneity. However, comparative simulations showed only a small effect of density difference on the flow distribution. Additionally, the investigation of flow homogeneity by looking at CA distributions is an indirect method and does not give direct insight into the underlying flow velocity distributions. This method as well as our calculated HI is only applicable when making a relative comparison between different specimens. Moreover, the CT scans of CA distribution were only performed once per specimen. We did not evaluate the repeatability of the measurements. Still, the repeatability of CT scanning with and without CA is reported high when the measurements protocol is kept identical, which was the case in our experiments.^[^ ^37^, ^38^, ^39^ ^]^ Finally, our investigation mainly focused on the manipulation of the TPMS lattice structure and its effect on fluid flow distribution. We did not manufacture actual gas transfer membranes but demonstrated possible membrane structure geometries. Therefore, we did not evaluate gas transfer efficiency, which is crucial for membrane oxygenators. However, we have reported an increase in gas transfer with increasing flow homogeneity, prior.^[^ ^12^ ^]^

## 4 Conclusion

In summary, we have introduced a method for the multi‐scale adaptation of TPMS lattice structures to establish smooth, optimized fluid flow distributions, tailored to specific requirements. Within our experimental validation, we have shown the effectiveness of our approach to improve fluid flow homogeneity. Our method can be applied for ECMO to design TPMS‐based membrane structures with enhanced hemocompatibility and mass transfer efficiency.^[^ ^9^, ^10^, ^11^ ^]^ This method can also be expanded toward the establishment of desired fluid flow parameters, like shear stress or local volume flow, that mimic physiological conditions to enhance biocompatibility of blood contacting devices in general or to improve cell adhesion, e.g., to promote surface functionalization by endothelialization.^[^ ^40^ ^]^ Moreover, the demonstrated pipeline for lattice structure manipulation is fully versatile and can be directly transferred to a wide range of technical applications, e.g. heat exchangers or membrane contactors. In these examples, a tailored flow with improved homogeneity would also lead to enhanced long‐term stability and increased efficiency due to less stagnation regions that promote fouling effects or do not contribute to heat or mass transport.

## 5 Experimental Section

### Implementation of Local Geometry Variation

The local adjustment of TPMS structure geometry was performed by the introduction of a coordinate‐transformation. Using this method, it keep the basic TPMS lattice geometry and only scale it in size. The transformation is explained in Equations ((1), (2), (3)). Here, the TPMS geometry was defined by implicit mathematical equations. In Equation (1), the implicit formulation for a Schwarz Diamond (SWD) TPMS geometry was given. Our study focuses on the SWD TPMS geometry because it has been identified as one of the most beneficial in terms of mass transfer efficiency while providing the highest surface area to volume ratio among different TPMS types.^[^ ^9^, ^10^ ^]^ Nevertheless, our proposed adaptation method works for all TPMS types. A scaling of the TPMS unit cell size can be achieved by modifying the argument of the sine and cosine functions within this equation with respect to the global coordinate axes.

(1) $$\cos\left(x\right)\cdot \cos\left(y\right)\cdot \cos\left(z\right)-\sin\left(x\right)\cdot \sin\left(y\right)\cdot \;\sin\left(z\right)=0$$

(2) $$\frac{\partial \vec{x_{i}}}{\partial \vec{X_{i}}}=\frac{1}{cs}\;\left(\begin{array}{c} 1\\ 1\\ 1 \end{array}\right)$$

(3) $$\vec{x}=\;\left(\begin{array}{c} x\\ y\\ z \end{array}\right)=2\pi \int_{0}^{{\vec{X}}_{i}}\frac{\partial \vec{x_{i}}}{\partial \vec{X_{i}}}\;d\vec{X_{i}}$$

First, from a local input value of unit cell size (*cs*), the gradient of the input of the implicit TPMS equation, $\vec{x}$, with respect to the global coordinate axes values, $\vec{X}$, is calculated from Equation (2) to achieve the desired scaling. This gradient must be set equally in all three coordinate directions to retain the triply periodic property of the surface structure. Every local change in TPMS unit cell size affects the argument of the implicit TPMS equation further down the coordinate system. Therefore, the absolute values of $\vec{x}$ for every spatial location were calculated by a cumulative integral (Equation 3) along every coordinate axis to ensure steadiness of the whole TPMS surface.

### Numerical Structure Optimization Algorithm

The automatic optimization algorithm of the TPMS structure was based on a porous medium CFD simulation. The immediate numerical simulation of flow inside the TPMS structure geometry, which was in the scale of micrometres, was not feasible for the whole oxygenator due to excessive computational demand. Therefore, microscopic simulations of a small section of the TPMS structure with 2×2×3 (X, Y, Z) unit cells were performed for different cell sizes and flow velocities, analogue to Barbian et al.^[^ ^12^ ^]^ Based on the microscopic simulation results, the hydraulic properties of the SWD TPMS structure were determined and described based on the Darcy‐Forchheimer formulation. Here, the hydraulic pressure loss, ∇*p*, was calculated by taking the combination of viscous losses (D) and momentum losses (F) into account (cf. Equation 4).^[^ ^41^ ^]^

(4) $$\nabla p=\left(\eta D+\frac{1}{2}\rho F\vec{u}\right)\;\vec{u}$$

where η is the fluid viscosity, ρ is the fluid density and $\vec{u}$ is the velocity. The respective Darcy and Forchheimer coefficients are shown in Figure S1 (Supporting Information) for different unit cell sizes. These characteristics were implemented into a steady state porous medium CFD model. All CFD simulations within our study were performed using CFX R2023 R2 (Ansys, USA). The macroscopic design for the oxygenator prototypes was derived from a CT scan of a commercial oxygenator (Maquet Quadrox‐I Small Adult). In‐ and outlet flow distributor geometries were kept while the outer and inner distributor plates from the QSA were omitted when designing the TPMS‐based prototypes. The outer dimensions of the TPMS structure region were kept identical to those of the HFM modules inside the QSA.

**Figure 8:**
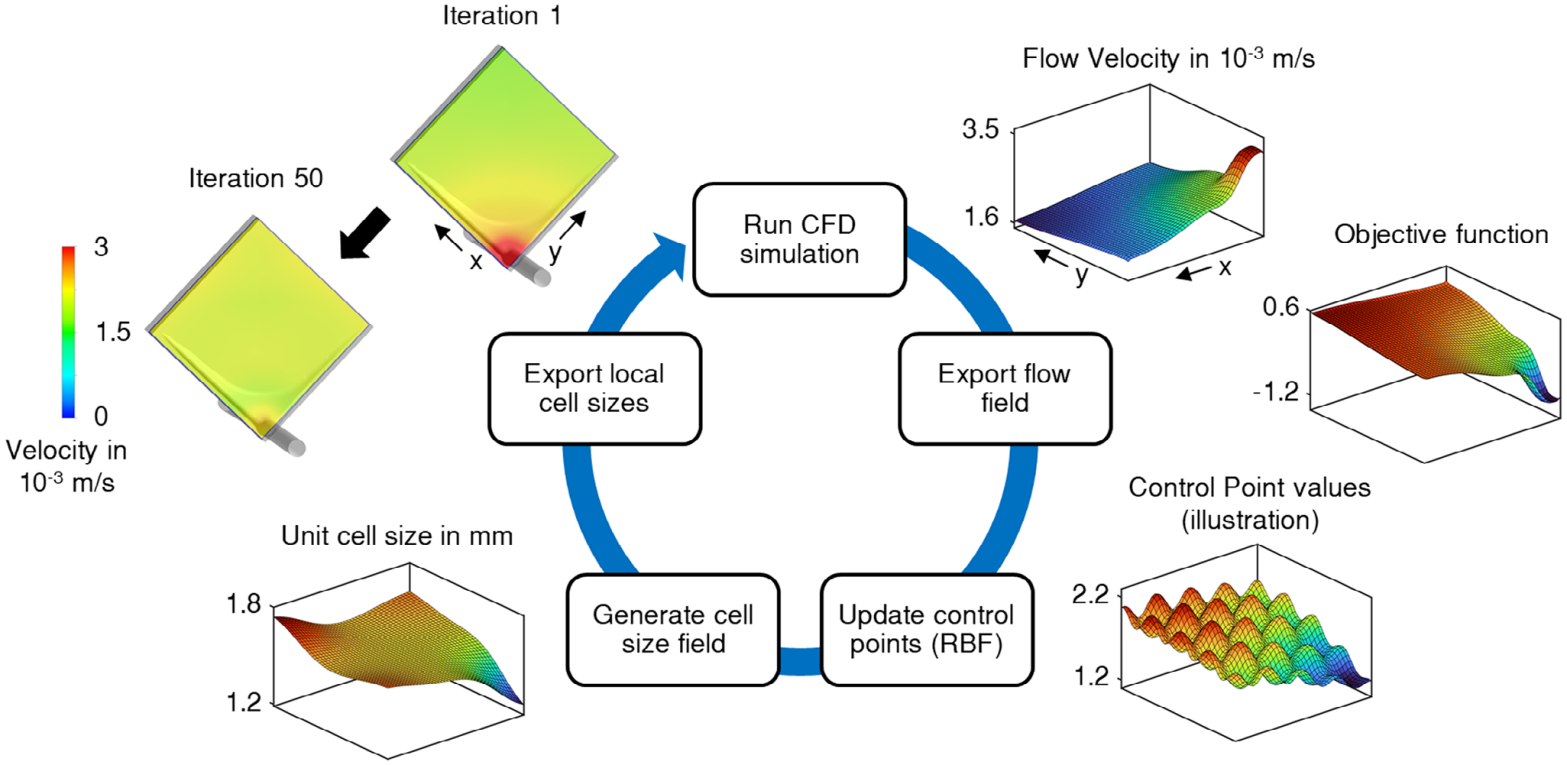
Steps of the automatic optimization algorithm for the generation of the adapted, anisotropic TPMS unit cell size field.

The automatic structure optimization was performed in Matlab (R2023b). The aim of the optimization was to yield a scalar field of TPMS unit cell sizes that provides homogenous flow velocity distribution inside the membrane structure. First, the TPMS unit cell size was initialized with a value of 1.2 mm, which was the smallest size that can be reliably manufactured with our in‐house equipment. During the optimization process, the local unit cell sizes were defined using a grid of control points. Radial basis functions with a Gaussian kernel were used to smoothly merge the local control point input with its surrounding unit cell size field (cf. **Figure** **8**). The optimization problem was defined to minimize the magnitude of the difference between local flow velocity and a reference velocity that was calculated as the total volume flow divided by the cross‐section area of the TPMS structure and the porosity. Each optimization iteration consisted of the following steps: First, from the results of the CFD simulations, the input to the objective function (Equation 5) was evaluated. Second, the values of unit cell size for all control points were updated based on the optimization rule (Equation 6).

(5) $$minimize:abs\left(u-u_{ref}\right)$$

(6) $$c\;s_{i+1}=\max\left(cs_{min},cs_{i}-\alpha \left(u-u_{ref}\right)\right)\;$$

where cs is the unit cell size, α is the optimization rate and u is the flow velocity magnitude. Based on the new unit cell size values of the control points, a cell size field was generated and smoothed in a subsequent step. This unit cell size field was then exported to the CFD simulation and used as input for the following simulation. The optimization steps are illustrated in Figure 8. This optimization loop was performed for a minimum of 50 iterations and until a termination criterion was met. A change in unit cell size value at every location that was less than 5% between two iterations was demanded as termination criterion.

### Test Specimen Manufacturing

Based on the optimized cell size field, two TPMS lattice structures were manufactured for experimental testing. For the first structure, the optimized cell size field was converted into the TPMS geometry directly, using the transformation method described in Equations ((1), (2), (3)). The second structure consisted of a constant TPMS unit cell size of 1.79 mm that yields the same membrane surface area as the optimized version. One major challenge was the transformation of the unit cell size fields into a geometry representation that can be interpreted by a 3D printing environment. To achieve this goal, it created a transformation script in Python (v3.10) that makes use of implicit geometry representation.^[^ ^42^ ^]^ Based on Equations ((1), (2), (3)), a 3D signed distance field (SD) was calculated (Equation 7):

(7) $$SD=\cos\left(x\right)\;\cdot \cos\left(y\right)\cdot \cos\left(z\right)-\sin\left(x\right)\cdot \sin\left(y\right)\cdot \sin\left(z\right)-c$$

where SD is the scalar signed distance value and c is a membrane surface translation variable that was used for fine tuning of manufacturing accuracy. The geometry for the 3D print was then extracted by thresholding the SD field where a numerical value of 0 or less represent the regions to be filled with material. With a spatial resolution of 16.8 × 24.8 × 25 µm (X × Y × Z), representing the 3D printer voxel resolution, this transformation process was executed using explicit message passing interface parallelization on the RWTH high performance compute cluster on 140 processors demanding 1.81 TB of working memory.^[^ ^43^ ^]^ The output of this translation process was a black and white image stack in combination with a gcode file containing instructions for the 3D slicing software on how to reconstruct the voxel geometry. After this export, the geometry was resliced using Chitubox Basic (v2.1) to create the proprietary file format for printing. For the 3D model visualization of the structure in Figure 3c,d, a similar approach to the generation of the 3D printing file was used. Instead of the black and white image stack, 128‐bit images were created in the DICOM format using the pydicom library to include information about spatial coordinates for reconstruction. These image files were imported into and visualized in 3D Slicer via a threshold segmentation.^[^ ^44^ ^]^ The 3D printing was performed on an Anycubic Photon Mono M5s pro machine that uses masked stereolithography in combination with the Phrozen Aqua 8k photopolymer. After printing, the TPMS structures were flushed and cleaned in an ultrasonic bath with isopropanol for 20 min. Afterwards, the parts were dried in an oven at 70 °C for 2 h and post cured under UV light for 4 h. The accessory parts containing inlet and outlet distributors were also 3D printed as composite materials including an elastomeric sealing on a Stratasys Objet Eden 350 Polyjet printer.

### Experimental Assessment of Flow Distribution

**Figure 9:**
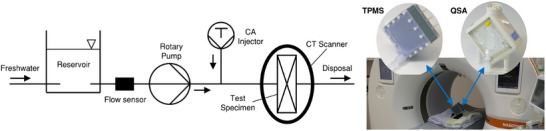
Experimental testing setup for the time‐resolved CT‐measurements of CA distribution.

To evaluate the homogeneity of fluid flow through the different structures, it carried out time resolved, contrast‐enhanced CT scans with a state‐of‐the‐art, photon‐counting scanner (Siemens Naeotom Alpha). With this photon‐counting technology, X‐ray photons were detected directly by sensors whilst measuring their quantity and quantum energy. Therefore, more information was available for image reconstruction, which leads to increased image quality through less noise and reduced artefacts.^[^ ^45^ ^]^ Sequential scans were performed without table feed using the full detector width. Prior to the scans, it established a constant, stationary flow of water through the specimens using a rotary pump (Deltastream DP3, Fresenius Medical Care, Germany) and an ultrasonic flow sensor (ME9PXL, Transonic, USA). With the beginning of the scan, a short bolus of iodine CA (Ultravist 370, Bayer, Germany) was injected into the flow ahead of the specimen inlets using an automatic injection system (FlowSens, Medex, France) for a time of 2 s. During the measurement time of ≈30 s, the distribution of the CA was recorded over time with a temporal resolution of 0.5 s. Scan parameters and were provided in the supplementary material (Table S1, Supporting Information) Measurements were performed for both TPMS structures, constant and optimized, and the commercial oxygenator for water flow rates of 0.5 and 1.0 l min^−1^ each in combination with CA flow rates of 2 and 4 mL s^−1^, respectively. Thereby, six measurements were performed in total. The measurement setup is displayed in **Figure** **9**.

Prior to these measurements, static CT scans of pure CA and all specimens in dry condition were performed with otherwise identical scan parameters. With the imaging data of the specimens’ flow channels being filled with air, with water and with the transient measurement data of CA perfusion, the local CA concentrations can be calculated (Equation 8):

(8) $${\vec{\Phi }}_{CA,t}=\frac{{\vec{HU}}_{t}-\left(1-\vec{\varepsilon }\right)\cdot \frac{HU_{material}}{\vec{\varepsilon }}-HU_{water}}{\left(HU_{CA}-HU_{water}\right)}\;$$

(9) $$\vec{\varepsilon }=\frac{{\vec{HU}}_{t0}-\;{\vec{HU}}_{air}}{\left(HU_{water}\;-HU_{air}\right)}\;$$

where ${\vec{\Phi }}_{CA,t}$ is the CA concentration field, ${\vec{HU}}_{t}$ is the measured Hounsfield unit (HU) field of the CT scans and $\vec{\varepsilon }$ is the volume porosity field of the individual structures. The variable HU with the indices material, water, CA and air represent a constant, known HU value of the respective substances. The local volume porosity field $\vec{\varepsilon }$ was calculated separately according to Equation 9. This calculation makes use of the assumption of a homogenous distribution of membrane material and void of the flow channels inside each voxel and amongst all voxels. For this assumption to hold, a down sampling of the CT scan results was performed by taking the arithmetic average over multiple neighboring voxels. The data was down sampled to a voxel size with 2 mm edge length. From this insight into the local CA concentrations, the qualitative spatial CA distributions inside all specimens were evaluated for different time points. Moreover, the average CA concentrations in specific regions of the structures were extracted over time. Visualization was performed in Paraview (Kitware, USA). Finally, a homogeneity index (HI) was calculated as a quantitative metric for homogenous perfusion of the structures. This index was defined as the arithmetic mean of standard deviations of CA concentration on different planes, which were perpendicular to the main flow direction (Equation 10).

(10) $$HI=1-\frac{1}{SD_{max}}\cdot \frac{1}{n}\sum_{i=1}^{n}\sqrt{\frac{1}{m-1}\sum_{j=1}^{m}{\left(\Phi_{CA,j}-\bar{\Phi_{CA}}\right)}^{2}}$$

where SD_max_ is the maximum expected standard deviation, n is the number of cross‐sectional planes and m is the number of evaluation points per plane. When a homogenous mixing of CA and water was assumed before the inlet of the specimens, the maximum CA concentration that can occur was 20%. Therefore, the maximum standard deviation was estimated to be 10%. Thereby, the HI was mapped to a value between zero and one with zero indicating the least possible homogeneity and one the best homogeneity that was present when the CA concentration value was equal across all observation points.

### Validation of Numerical Simulations

For the validation of the numerical simulations, the experimental measurement setup was recreated in silico. Transient simulations of the three specimens were performed using a homogenous two‐phase model for the water‐CA interaction and the porous medium simplification.^[^ ^46^ ^]^ The porous medium characteristics for TPMS structures were adapted from the simulations of the optimization algorithm. The parameters of the HFM structure inside the QSA was taken from Schlanstein et al.^[^ ^47^ ^]^ Buoyancy, based on density difference, was also considered. The simulations were run on the RWTH high performance compute cluster with each simulation demanding ≈500 core‐hours. Afterwards, the simulations were compared to the experimental results qualitatively in terms of spatial CA distributions and quantitatively looking at the average CA concentration and the HI curves over time. Moreover, a Spearman's correlation analysis of region averaged CA concentration at all time points was performed.

### Statistical Analysis

A statistical analysis was carried out for correlation evaluation in chapter 2.4. Coordinate transformations were performed to match the grids of experiment and simulation. The spatial CA concentration was calculated according to Equations ((8), (9), (10)). The CA concentration was averaged to create a uniform grid of 147 data points per time step. All the timesteps from the experiment were included in the data set for the correlation analysis summing up to 9849 data points. The data was checked for the number of tied ranks to be below 1%. A Spearman correlation test was performed in Matlab (R2023b) to yield correlation coefficients with p‐values below 1e‐3.

## Conflict of Interest

The authors declare no conflict of interest.

## Supporting information



Supporting Information

## Data Availability

The data that support the findings of this study are available from the corresponding author upon reasonable request
