# Supplementary material for: Tailored 3D Lattice Microstructures for Enhanced Functionality in Blood‐Gas Exchange
Source: Adv Sci (Weinh). 2025 Apr 17;12(25):2501162. doi: 10.1002/advs.202501162 (PMC12224992; doi:10.1002/advs.202501162)
Supplement: Supplementary file 1 — Supporting Information [file ADVS-12-2501162-s001.docx]

Supporting Information

Tailored 3D Lattice Microstructures for Enhanced Functionality in Blood-Gas Exchange

Kai P. Barbian*, Teresa Lemainque, Ina Grunden, Roman Iwa, Bettina Wiegmann, John Linkhorst, Matthias Wessling, Jan Heyer, Ulrich Steinseifer, Michael Neidlin
and Sebastian V. Jansen

Figure S1: Calculated hydraulic properties of viscous loss coefficient (D, left) and momentum loss coefficient (F, right) for different SWD TPMS unit cell sizes and for water with constant dynamic viscosity and blood including shear thinning behavior (Carreau-Yasuda model).


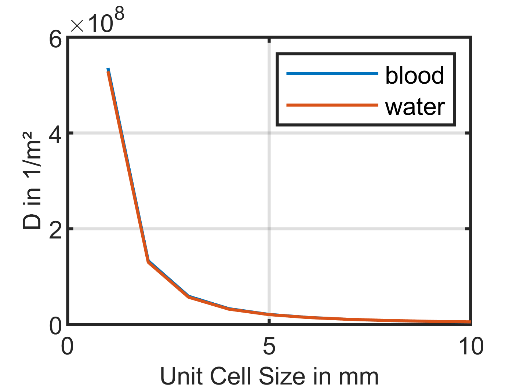

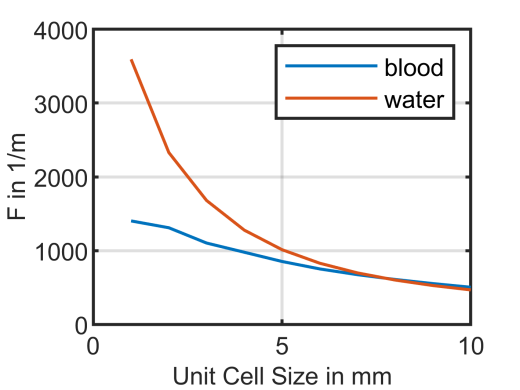


Table S1: CT scan parameters, KVp: Kilovolt peak, rpm: rotations per minute.

| Scanner model | Siemens Naeotom Alpha |
| --- | --- |
| Scanning protocol | Lung embolism |
| KVp | 140 kV |
| Tube current in | 279 mA |
| Convolution kernel | Br36f |
| Reconstruction pixel spacing | 0.4165, 0.4165 mm |
| Reconstruction slice thickness | 0.6 mm |
| Gantry rotational speed | 240 rpm |
| Collimation width | 57.6 mm |


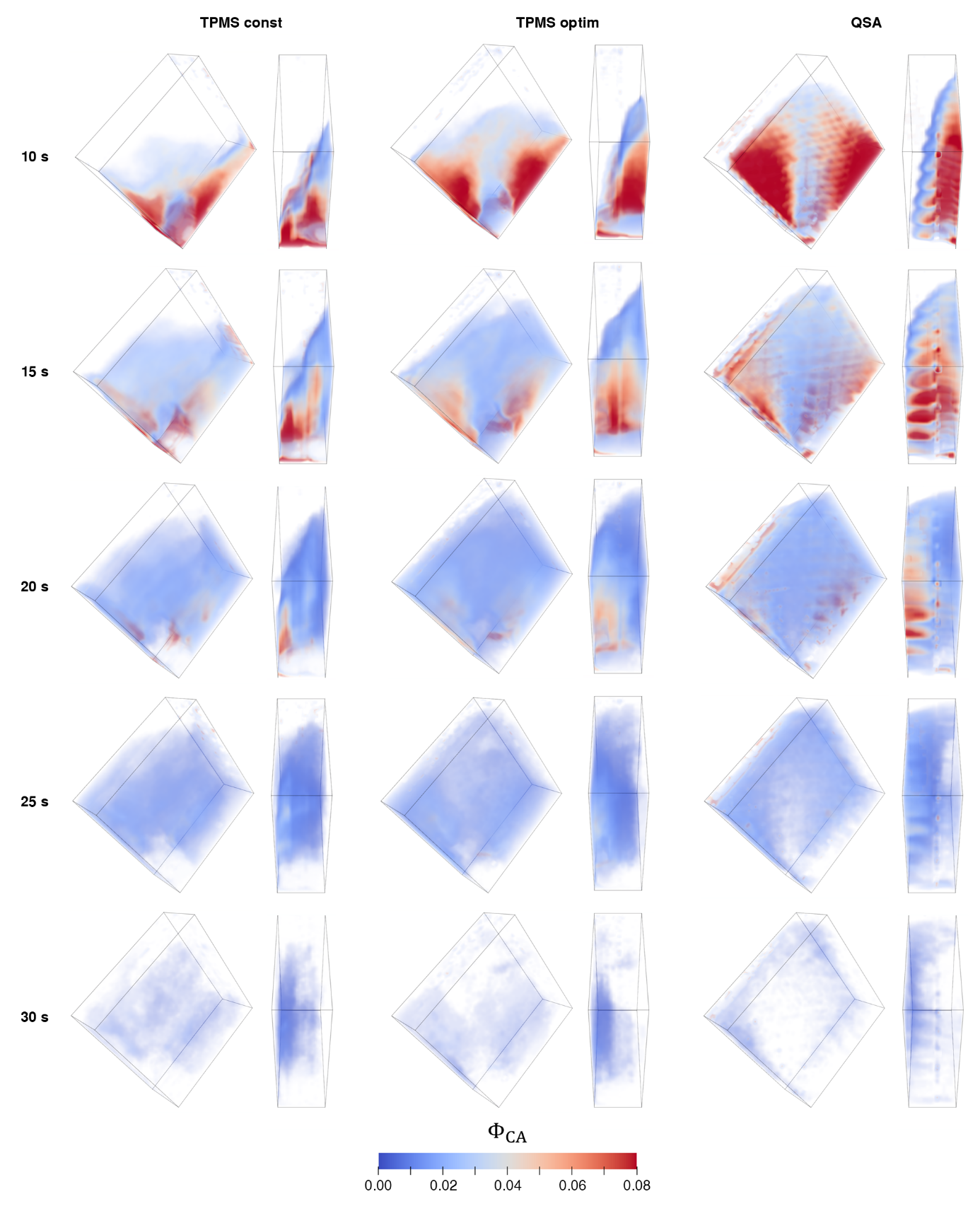


Figure S2: Spatial CA concentration distributions at different time steps for all test specimens, evaluated from the CT measurements at a flow rate of 0.5 l/min (extended).

Figure S3: Spatial CA concentration distributions at different time steps for all test specimens, evaluated from the CT measurements at a flow rate of 1.0 l/min (extended). Measurements of QSA are not representative here, because of the presence of trapped air inside the top of the central separation grid.


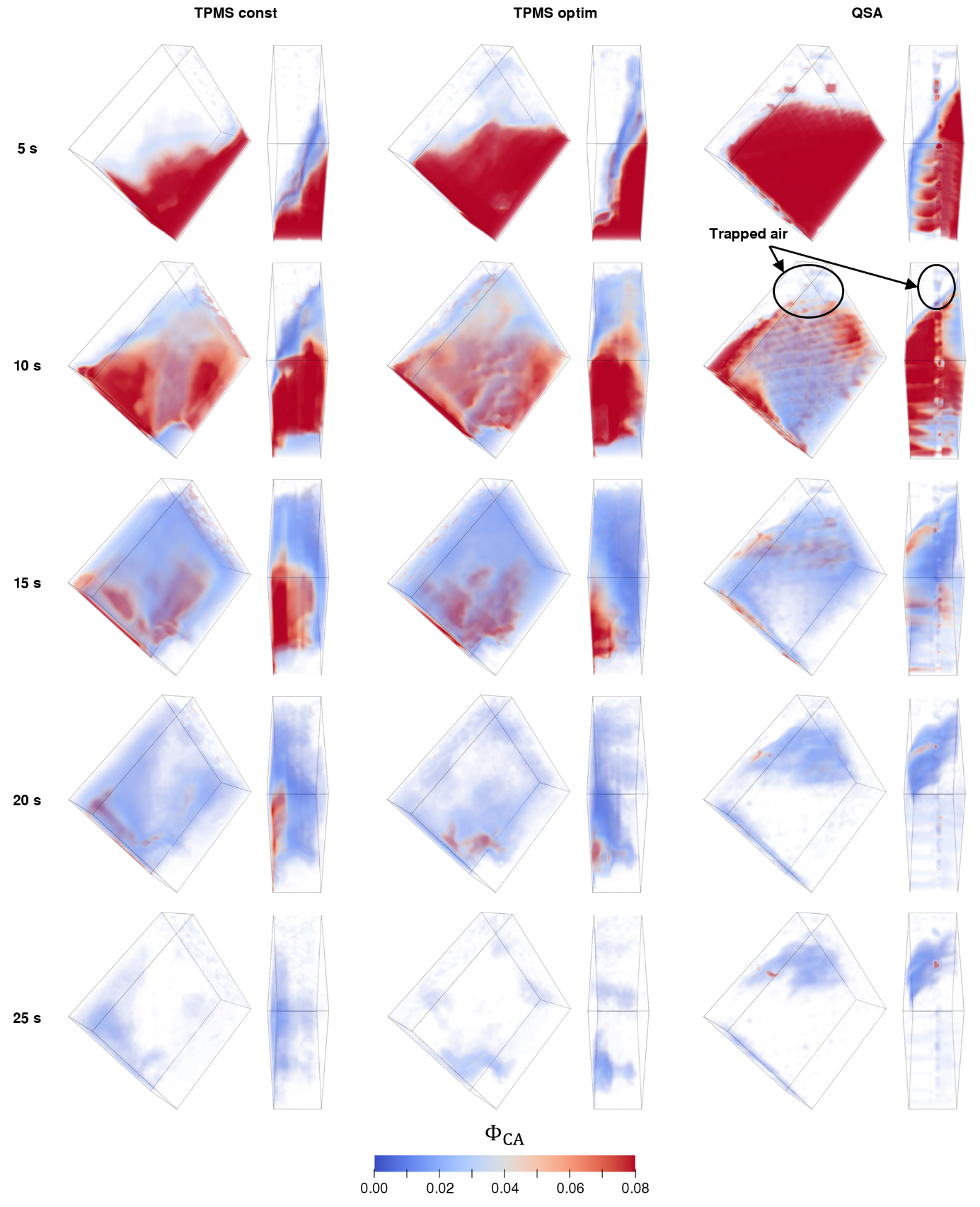


Figure S4: Region-averaged CA distributions and homogeneity indices over time from experiment at 1.0 l/min flow rate. CA distribution: first three graphs from the left. Blue line: CA concentration in bottom corner area, red line: left corner area, yellow line: center area, purple line: right corner area, green line: top corner area. Homogeneity indices: right graph. Black line: TPMS structure with constant unit cell sizes, blue line: TPMS structure with optimized unit cell size distribution, red line: commercial reference oxygenator.


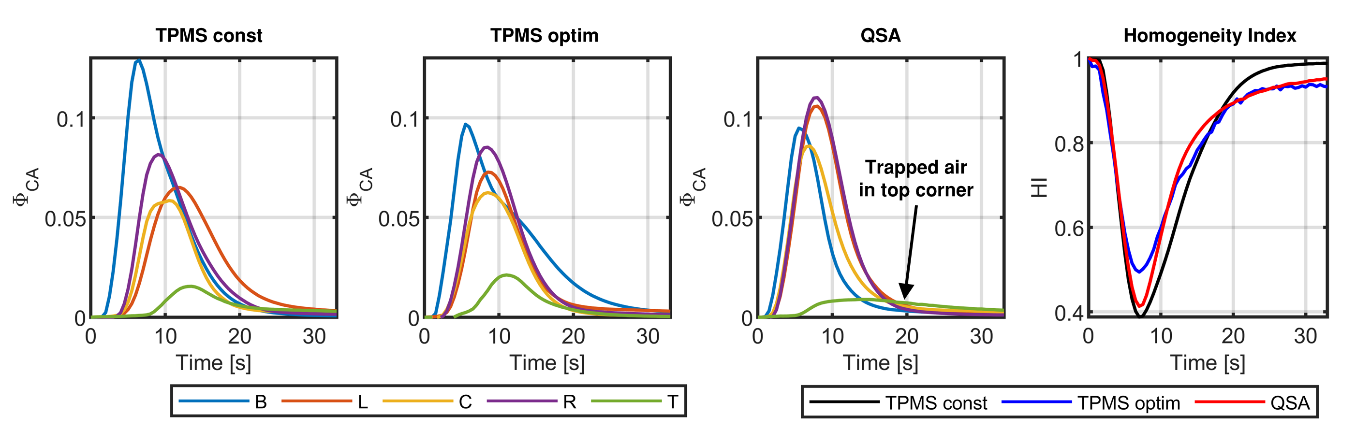

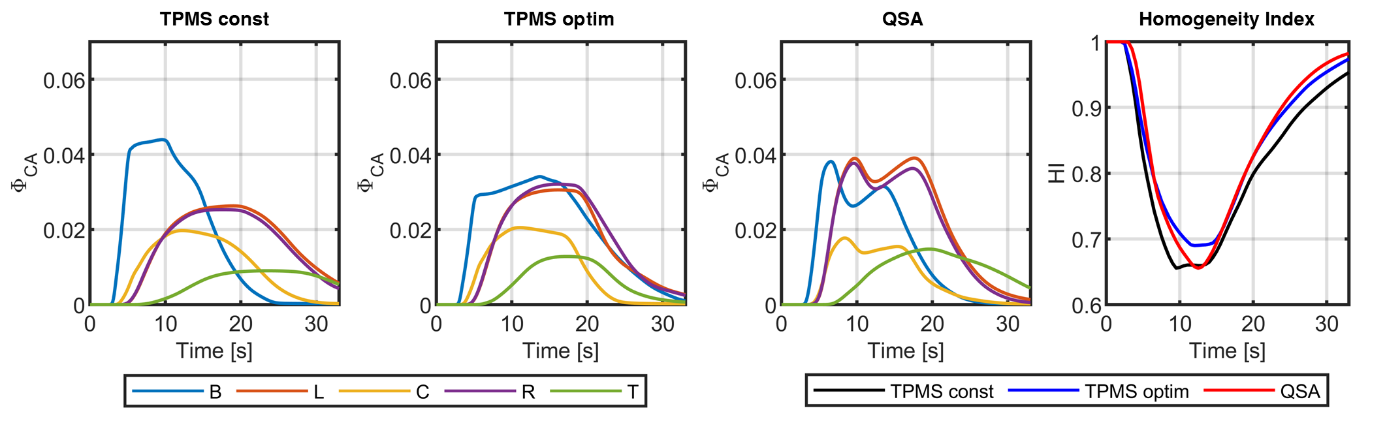


Figure S5: Region-averaged CA distributions and homogeneity indices over time from simulations at 0.5 l/min flow rate. CA distribution: first three graphs from the left. Blue line: CA concentration in bottom corner area, red line: left corner area, yellow line: center area, purple line: right corner area, green line: top corner area. Homogeneity indices: right graph. Black line: TPMS structure with constant unit cell sizes, blue line: TPMS structure with optimized unit cell size distribution, red line: commercial reference oxygenator.


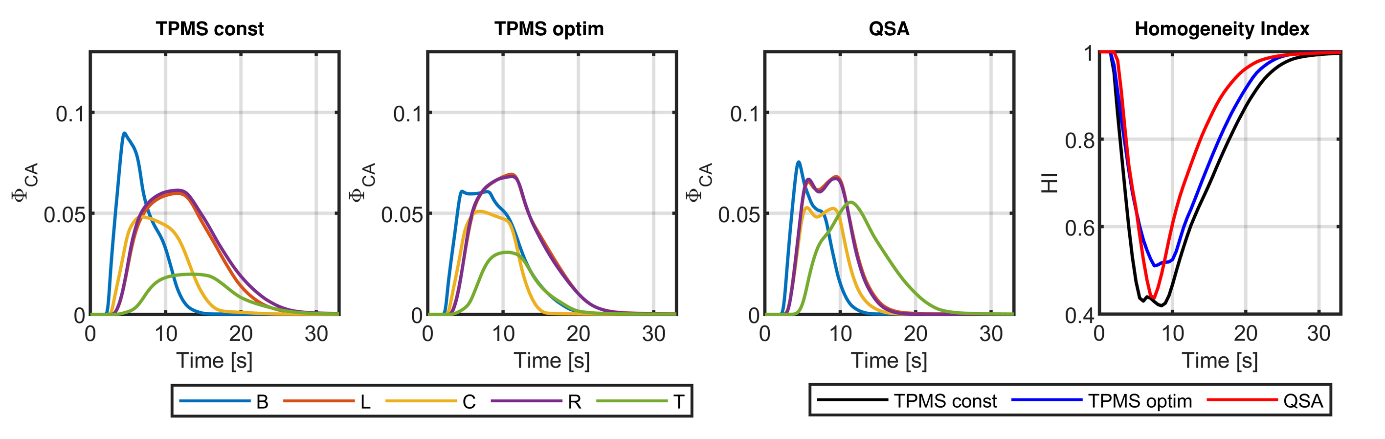


Figure S6: Region-averaged CA distributions and homogeneity indices over time from simulations at 1.0 l/min flow rate. CA distribution: first three graphs from the left. Blue line: CA concentration in bottom corner area, red line: left corner area, yellow line: center area, purple line: right corner area, green line: top corner area. Homogeneity indices: right graph. Black line: TPMS structure with constant unit cell sizes, blue line: TPMS structure with optimized unit cell size distribution, red line: commercial reference oxygenator.


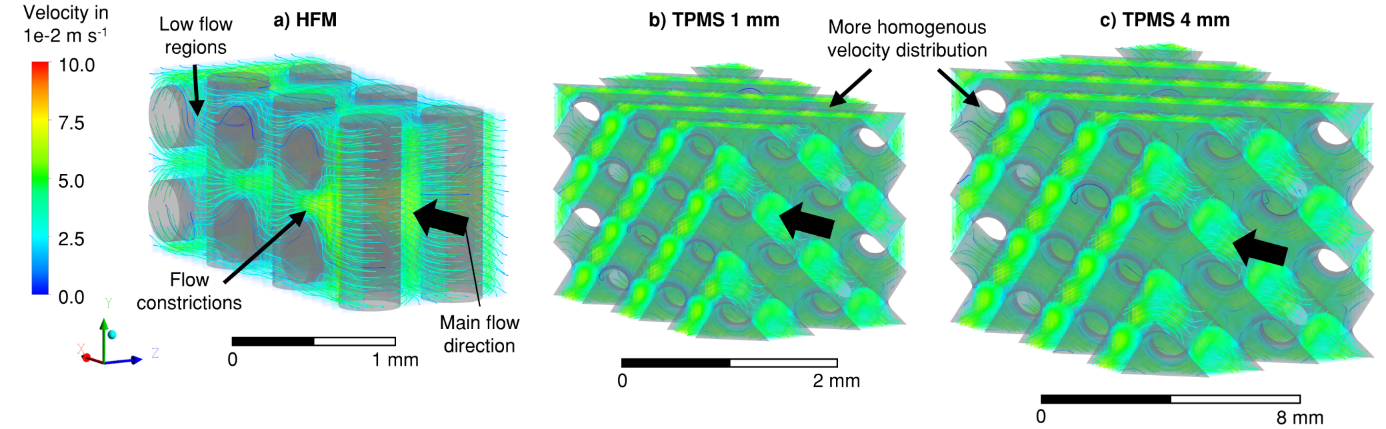


Figure S7: Velocity streamlines inside 3x2x2 (XxYxZ) periodic elements of different microstructure geometries.^[12]^ a) stacked HFM, b) 1 mm SWD TPMS unit cells, c) 4 mm SWD TPMS unit cells. The TPMS structure provides increased passive mixing and more homogenous distribution of flow velocity magnitude which is preserved within scaling.
